# Supplementary material for: Stress-dependent miR-980 regulation of Rbfox1/A2bp1 promotes ribonucleoprotein granule formation and cell survival
Source: Nat Commun. 2018 Jan 22;9:312. doi: 10.1038/s41467-017-02757-w (PMC5778076; doi:10.1038/s41467-017-02757-w)
Supplement: Supplementary file 2 — Supplementary Information [file 41467_2017_2757_MOESM2_ESM.pdf]

Supplementary Table 1. *miRNA-980* expression measured by qRT-PCR

Experiment	Genotype	C_T (*2S*), AVE±SD	C_T (*miR-980*), AVE±SD	ΔC_T = C_T (*miR-980*) – C_T (*2S*), AVE±SD	$\Delta\Delta C_T$ = ΔC_T (Experiment) - ΔC_T (Control), AVE±SD	Relative *miR-980* expression, AVE±SD
**LOF** (*Exp. 1*)	Control *FM7/+* (males)	7.08±0.23	29.92±0.03	22.83±0.26	0.00±0.26	1.01±0.19
	miR-980^{Ex2} (males)	6.80±0.20	35.00±0.31	28.20±0.32	5.36±0.32	0.02±2.8x10^{-3}
	miR-980^{Ex1-2} (males)	6.72±0.17	38.99±0.95	32.27±1.06	9.43±1.06	1.70x10^{-3} ± 8.00x10^{-4}
	miR-980^{Ex1-3} (males)	6.79±0.18	36.82±0.29	30.03±0.35	7.80±0.35	6.91x10^{-3}± 1.70x10^{-3}
**LOF** (*Exp. 2*)	Control *FM7/+* (males)	5.42±0.07	23.67±0.03	18.30±0.08	0.00±0.08	1.00±0.05
	miR-980^{Ex1-1} (males)	6.96±0.08	38.99±0.95	32.27±1.06	9.43±1.06	1.7x10^{-3}± 0.98x10^{-3}
**LOF** (*Exp. 3*)	Control *OregonR/w^{1118}* (ovaries)	6.40±0.16	32.38±0.04	25.98±0.04	0.00±0.04	1.00±0.03
	miR-980$^{KO/Ex1-2}$ (ovaries)	6.45±0.37	39.13±0.93	32.89±0.75	6.91±0.75	9.00x10^{-3} ± 5.20x10^{-3}
**LOF** (*Exp. 4*)	Control *w^{1118}* (ovaries)	5.17±0.05	32.61±0.07	27.44±0.07	0.00±6.13x10^{-6}	0.99±4.2x10^{-6}
	miR-980^{Ex1-3} (ovaries)	5.17±0.02	37.56±0.10	32.39±0.10	4.90±0.72	0.03±5.4x10^{-3}
GOF	Control *OregonR* (females)	6.53±0.08	31.83±0.16	25.33±0.13	0.00±0.13	1.00±0.09
	Rbfox1Gal4/ UAS-miR-980 (females)	6.24±0.03	30.08±0.23	24.56±0.27	-0.77±0.36	1.74±0.44
Stress response	Control *w^{1118}* (males) Normal food, 25°C	5.92±0.16	27.40±0.05	21.48±0.11	0.00±0.11	0.99±0.08
	w^{1118} (males) Protein deficit, 25°C	5.99±0.12	29.39±0.16	23.40±0.24	1.92±0.24	0.26±0.04
	w^{1118} (males) Sugar deficit, 25°C	5.49±0.13	27.53±0.13	22.04±0.19	0.55±0.19	0.68±0.09
	w^{1118} (males) Normal food, 33°C	5.33±0.11	27.39±0.19	22.06±0.14	0.58±0.14	0.67±0.07

Supplementary Table 2. Luciferase activity assays show that the extended Rbfox1 *3'UTR* can be targeted by the *miR-980* miRNA

miRNA expression plasmid	psiCHECK-2 plasmid	Renilla/Firefly luciferase ratio[a]	Relative luciferase levels[b]	p-value
none	*psiCHECK-2*	0.31±0.01	1.00±0.01	-
	psiCHECK-2-Rbfox1-3'UTR-P1	0.14±0.02	1.00±0.12	-
	psiCHECK-2-Rbfox1-3'UTR-P2	0.07±0.01	1.00±0.10	-
miR-980	*psiCHECK-2*	0.36±0.03	1.00±0.09	-
	psiCHECK-2-Rbfox1-3'UTR-P1	0.09±0.01	0.53±0.10	**p=0.003[c] *p=0.023[d]
	psiCHECK-2-Rbfox1-3'UTR-P2	0.04±0.01	0.54±0.10	**p=0.003[c] *p=0.018[d]
miR-966	*psiCHECK-2*	0.38±0.02	1.00±0.04	-
	psiCHECK-2-Rbfox1-3'UTR-P2	0.09±0.02	0.98±0.04	p=0.899[c] p=0.223[d]
miR-278	*psiCHECK-2*	0.42±0.08	1.00±0.04	-
	psiCHECK-2-Rbfox1-3'UTR-P2	0.07±0.01	0.73±0.17	p=0.101[c] p=0.665[d]

The Firefly luciferase expression is used as an endogenous control where 2 different parts of extended *Rbfox1 3'UTR* were cloned into the *Renilla luciferase* gene.
[a] Renilla to Firefly luciferase luminescence value ratios with the background subtracted;
[b] values in the presence of miRNA were normalized to the values measured with no miRNA-expressing plasmid (none) and to the values of plasmid without *Rbfox1-3'UTR* regions (*psiCHECK-2*);
[c] comparison of values obtained from *Rbfox1-3'UTR-P1* and *Rbfox1-3'UTR-P2* with *psiCHECK-2* in the miRNA presence;
[d] comparison of values obtained from *Rbfox1-3'UTR-P1* and *Rbfox1-3'UTR-P2* with and without a miRNA;
AVE±SD are reported from experiments done in triplicate and significance was tested using a two-tailed Student's t-test; *p≤0.05; ** p≤0.01

Supplementary Table 3. *Rbfox1* and *CG3777* mRNA levels measured by qRT-PCR

	Genotype	C_T (*Rpl32*), AVE±SD	C_T (*Rbfox1 or CG3777*), AVE±SD	ΔC_T = C_T (*Rbfox1 or CG3777*) – C_T (*Rpl32*), AVE±SD	$\Delta\Delta C_T$ = ΔC_T (Experiment) - ΔC_T (Control), AVE±SD	Relative mRNA levels, AVE±SD
***Rbfox1* expression**	Control, w^{1118} (ovaries, normal food)	14.01±0.05	24.04±0.03	10.03±0.03	0.00±0.03	0.99±0.02
	w^{1118} (ovaries, protein deficit)	13.82±0.14	22.85±0.05	9.04±0.05	-0.99±0.05	2.20±0.23
	Control, w^{1118} (ovaries, normal food)	12.77±0.49	24.62±0.03	11.84±0.03	0.00±0.03	1.00±0.02
	miR-980^{Ex1-3} (ovaries, normal food)	13.64±0.13	24.69±0.22	11.05±0.22	-0.79±0.22	1.74±0.26
***CG3777* expression**	Control, *OregonR* (males)	21.62±0.10	24.67±0.10	3.04±0.10	0.00±0.10	1.00±0.07
	Control, *FM7/+* (males)	22.35±0.09	25.31±0.08	2.96±0.08	-0.08±0.08	1.05±0.06
	miR-980^{NP3544} (males)	21.79±0.06	24.65±0.22	2.86±0.22	-0.18±0.22	1.14±0.18
	miR-980^{Ex1-1} (males)	21.57±0.10	24.72±0.26	3.15±0.26	0.11±0.26	0.92±0.17
	miR-980^{Ex1-2} (males)	22.82±0.04	25.67±0.09	2.90±0.01	-0.14±0.01	1.10±0.01
	miR-980^{Ex1-3} (males)	21.89±0.11	24.84±0.09	2.85±0.09	-0.18±0.09	1.14±0.07

[a] - the ΔC_T value is determined by subtracting the average C_T value of endogenous control gene (2S for miRNAs or Rpl32 for mRNA) from the average miRNA or mRNA C_T value.
[b] - the calculation of $\Delta\Delta C_T$ involves subtraction by the ΔC_T calibrator value (ΔC_T value in w^{1118}).
[c] - the range is given for relative levels determined by evaluating the expression: $2^{-\Delta\Delta CT}$.
AVE±STDEV values are reported from experiments done in triplicates. Two-tailed Student's t-test was used to test for statistical significance

Supplementary Table 4. Homology between *Drosophila* Rbfox1 and human RBFOX family proteins that are annotated in *Flybase*

Species	Gene name	Protein sequence ID	FlyBase/HGNC ID	Protein length (aa)	Total sequence identity	Total sequence similarity	Total gaps	Identity in RRM domain	Presence of LCD domains
D. melanogaster	***Rbfox1/ A2bp1***	NP 001246707.1	FBgn0052062	962	-	-	-	-	Yes
H. sapiens	***RBFOX1/ A2bp1***	NP 665898.1	18222	418	26 %	31 %	51 %	92 %	Yes
	RBFOX2	NP 001076047.1	9906	451	26 %	33 %	46 %	92 %	Yes
	RBFOX3	NP 001076044.1	27097	459	29 %	36 %	44 %	87 %	Yes

Supplementary Table 5. Low complexity sequence domains in *Drosophila* Rbfox1 and human RBFOX family proteins

Protein	Isoforms	Sequences of predicted LCD domains[1] (presented in N to C order)
***Drosophila* Rbfox1/ A2bp1**	E, F, L, J, K, H, I	VQAGVAPFPGAPAGYAAAPNPGAAVVAAAAAAQQQQQQQQQQQQQQQAQQQQQQVAGGPPSAADSLSMAVAAAAAKQSA
		SGSEAAGSGNSNNNNTAGAGTGAPGAAGGLTT
		GGCGGGGASTANSVVVATSVSDVVNAS
	J, K, H, I	TQTQTQYEYEYEY
		QVQQPPVQQQHLQSSLPPQ
	E, F, L, J, K, H, I, M	ANEAAESQQSSAMQNAGGGGNTGGGGGGGGGTPSSPLSNSPSSATAS
		PLLQTPPAHQQQQQQQPLLCSSSPTSMQSSGTSVTGSSIASGTLAATSSSG
		SLSSALVPAQSVAAVAAASLDAKS
	E, F, L, J, K, H	AAVAAA
		AAAAAAAYAARLSAATGATQSPQTAAAAAAAASMAASANAANNAAA
	E, F, L, J, K, H	AAVAQQQQQQQQAVVQQQQQQVAAAAQQQHQQQQQQQQQAVQQQQAVQQQQQHQQQQQQQQQQQH
	E, L, K, H, I, M	QAQQQAYATAATTYTAVAARAAYGAAAAAAAQPALAGYAT
	I, M	AQAPSAVAGGTAATSPATAAAAAAHAAAAAAAT
		PPHTAVQAATPTAATP
**Human RBFOX1/ A2bp1**	1-5	TATQTDDAAPTD
		AATAAAAYRGAHLRGRGR
		AAAPPPPIPA
		GFYGADIYGGYAAYRYAQPTPATAAAY
	5	LPTPTTTHLLQPPPTAL
**Human RBFOX2**	6, 8	PGAGGDGADPG
	1-10	QPFTTIPFPPPPQNGIP
		GGAQTDGQQSQTQSSENSESKST
		TAATTAAA
	1-3, 5-6, 8, 10	GFYGADLYGGYAAYRYAQPATATAATAAAAAAAAY
	4, 7, 9	LLLQPQPPLLQPLQPL
		PTPTMPLPLPLAMELAL
**Human RBFOX3**	1-5	AQPYPPAQYPPPPQ
		PPPPHP
		PPPPPIP
	1-3	LAPCPLPPQQTPEPAYPT
	1-5	GFYGAEIYGGYAAYRYAQPAAATAAAY

[1]LCD prediction was done by SMART (*http://smart.embl-heidelberg.de/*). FlyBase (*http://flybase.org/*) and UniProt (*http://www.uniprot.org/*) were used as sources for *Drosophila* and human protein sequences, respectively

Supplementary Table 6. ***Drosophila*** **Rbfox1 and human RBFOX family proteins contain multiple predicted miRNAs binding sites**

		Predicted miRNA target sites within 3'UTR	Number of predicted target sites (both conserved and poorly conserved)
D. melanogaster	***Rbfox1 (A2bp1/CG32062)***	*miR-980*	3
		miR-87	3
		miR-375	1
		miR-10-3p/1006	6
		miR-1014	2
		miR-9	2
		miR-987	1
		miR-960	4
		miR-180	1
		miR-276	1
		miR-33	3
		miR-1000	1
		miR-310c	1
		miR-79	1
		miR-263b	2
		miR-315	2
H. sapiens	***RBFOX1***	*miR-25/32/92abc/363/363-3p/367*	6
		let-7/98/4458/4400	11
		miR-217	1
		miR-7/7ab	2
		miR-30abcdef/30abe-5e/384-5p	5
	RBFOX2	*miR-25/32/92abc/363/363-3p/367*	1
		miR-129-5p/129ab-5p	2
		let-7/98/4458/4500	1
		miR-9/9ab	2
		miR-383	1
		miR-29abcd	1
		miR-135ab/135a-5p	1
		miR-125a-5p/125b-5p/351/670/4319	1
		miR-19ab	1
		miR-130ac/301ab/301b/301b-3p/454/721/4295/3666	1
		miR-148ab-3p/152	1
		miR-200bc/429/548a	2
		miR-22/22-3p	1
		miR-34ac/34bc-5p/449abc/449c-5p	1
	RBFOX3	*miR-205/205ab*	1
		miR-129-5p/129ab-5p	6
		miR-7/7ab	1
		miR-200bc/429/548a	1

Based on the TargetScanFly Release 6.0 [1] and TargetScanHuman Release 7.0 [2] miRNA target prediction databases

Supplementary Table 7. ***Drosophila*** **strains used in this study**

Gene affected	Allele	Allele description	Reference/ Source	Notes
Oregon R	*wild type*	-	BDSC	used as control
white	*w*^{1118}	loss of function *w* mutant	BDSC	
miR-980	*miR-980*^{NP3544}	hypomorphic *miR-980* mutant, transposable element insertion	[3] DGRC	used for generation of *miR-980* loss of function alleles
	miR-980^{Ex2}	loss of function *miR-980* mutant, 64bp insertion	generated in this study	used as *miR-980* loss of function mutant
	miR-980$^{Ex1\text{-}1}$<br>*miR-980*$^{Ex1\text{-}2}$<br>*miR-980*$^{Ex1\text{-}3}$	loss of function *miR-980* mutants, which are independently generated strains with an identical 31bp insertion		
	miR-980^{KO}	loss of function *miR-980* mutant, deletion	[4] BDSC	
	UAS-miR-980	*miR-980* under UAS promoter	[5] BDSC	used for *miR-980* overexpression
Rbfox1	*Rbfox1*^{EN403}	hypomorphic *miR-980* mutant, transposable element insertion	[6] gift from M.Buszczak	used as *Rbfox1* hypomorphic mutant
	Rbfox1^{MI09677}	hypomorphic *miR-980* mutant, transposable element insertion	BDSC	
	Rbfox1^{CC00511} *(Rbfox1-GFP)*	Insertion of the protein trap construct into an intron of Rbfox1 gene allowing for Rbfox1-GFP protein fusion	[7] BDSC	used for Rbfox1 expression pattern analysis and FRAP experiment, referred as to Rbfox1-GFP
	Rbfox1^{RNAi}	Rbfox1 RNA interference construct under UAS promoter	VDRC	used for *Rbfox1* downregulation
	UAS-Rbfox1-RE	Rbfox1 PE isoform under UAS promoter	[8] gift from LS.Shashidhara	used for *Rbfox1* overexpression
parkin	*park*^{1}	transposable element insertion	[9] BDSC	used for metabolic stress analysis

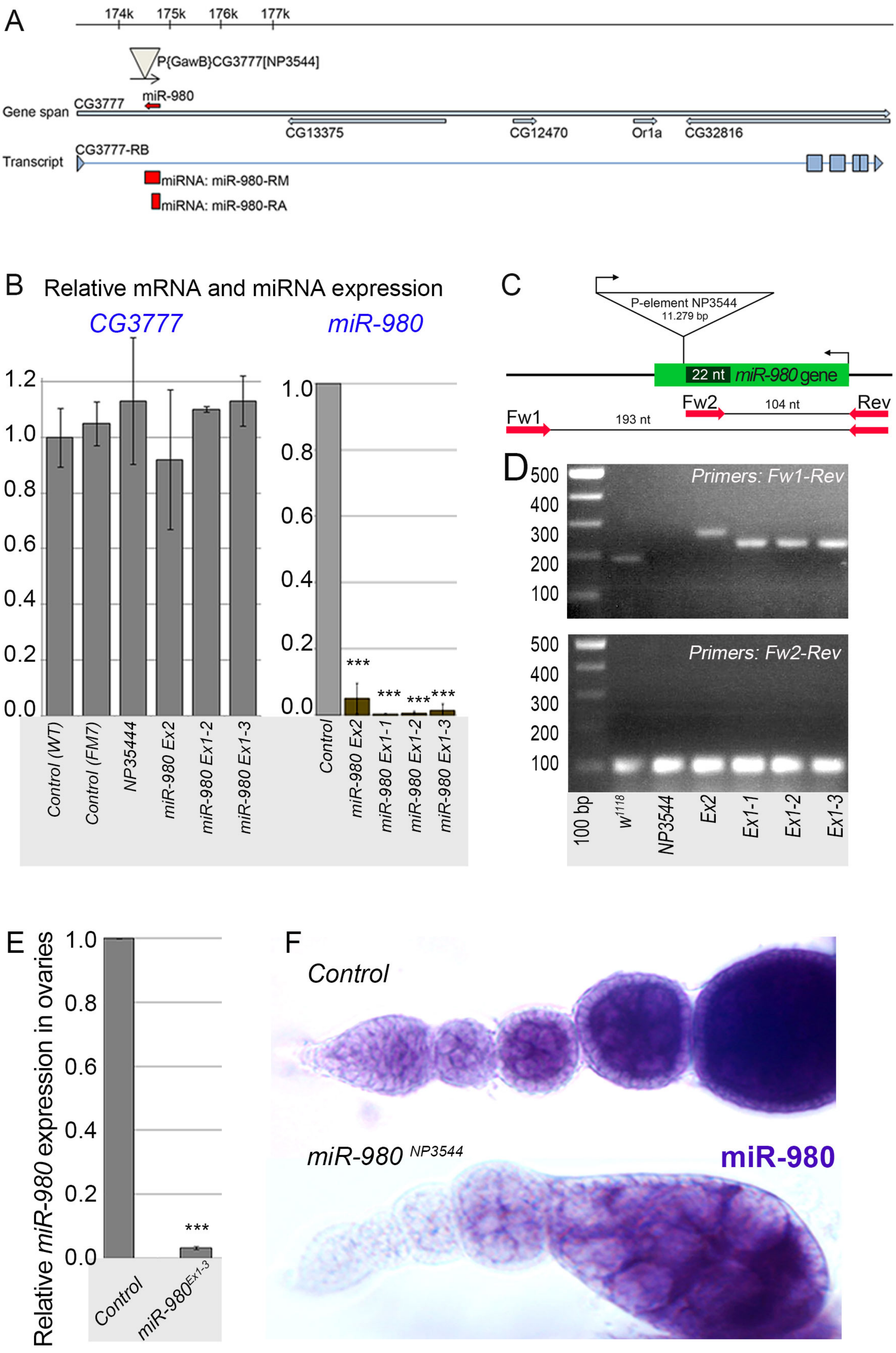

Supplementary Figure 1

Supplementary Figure 1. Generation and characterization of *miR-980* mutants

A, The gene encoding *miR-980* is located on the X-chromosome in a locus overlapping the first intron of *CG3777*, a gene of unknown function. A P-element insertion in *miR-980* results in a 70% reduction of mature *miR-980* levels [3] (here this hypomorphic allele is referred to as *miR-980*^{NP3544}). This P-element was mobilized in order to obtain *miR-980* loss of function mutants. **B**, Results of RT-qPCR analyses from whole males show that the mature *miR-980* is not produced in the newly generated *miR-980*$^{Ex1-1}$, *miR-980*$^{Ex1-2}$, and *miR-980*$^{Ex1-3}$ and *miR-980*^{Ex2} mutants, while no significant change in *CG3777* gene expression is detected (see also Tables S1 and S2). **C,** Scheme shows primer pairs used for mapping of *miR-980* mutants. **D,** Images of agarose gels show results of PCR amplification. In controls (*w*^{1118}), Fw1-Rev primers amplify a 193bp region, while in the parental *miR-980*^{NP3544} strain, no amplification is detected due to the presence of the large (11,279 bp) P-element insertion. Amplification products from *miR-980*^{Ex2}, *miR-980*$^{Ex1-1}$, *miR-980*$^{Ex1-2}$, and *miR-980*$^{Ex1-3}$ strains are increased in size when compared to *Control*, showing that the P-element was imprecisely excised. Note that the amplification product from *miR-980*^{Ex2} is larger than those from *miR-980*$^{Ex1-1}$, *miR-980*$^{Ex1-2}$, and *miR-980*$^{Ex1-3}$, which are of similar size. These data imply that *miR-980* mutants carry additional sequences left from the P-element. Sequencing results confirm that the *miR-980*^{Ex2} mutant contains a 64bp insertion (shown in brackets) in the *miR-980* gene: CCC[ATGATGAAATAACATATGTTATTTATGTATGTTATATGTTATATGTATAT GTTATTTCATCATG]CGTAAGCCCTTCACAAGGCAGCTAGCA, while *miR-980*$^{Ex1-1}$, *miR-980*$^{Ex1-2}$ and *miR-980*$^{Ex1-3}$ contain identical 31bp insertions at the same position: CCC[ATGATGAAATAACATATGTTATTTCATCATG]CGTAAGCCCTTCACAAG GCAGCTAGC. *miR-980*$^{Ex1-1}$, *miR-980*$^{Ex1-2}$, and *miR-980*$^{Ex1-3}$ strains were used for further analyses in this work.

E, Results of RT-qPCR analyses show loss of mature *miR-980* in ovaries in the newly generated *miR-980*$^{Ex1-3}$ mutant (see also Table S1). **F**, *miR-980* expression detected by LNA *in situ* hybridization is observed in both the somatic and the germline cells of *Drosophila* ovaries. The *miR-980*^{NP3544} hypomorphic mutant shows a decrease in *miR-980* expression.

AVE±STDEV values are reported from experiments done in triplicates. Two-tailed Student's t-test was used to test for statistical significance. ***$p \leq 0.001$.

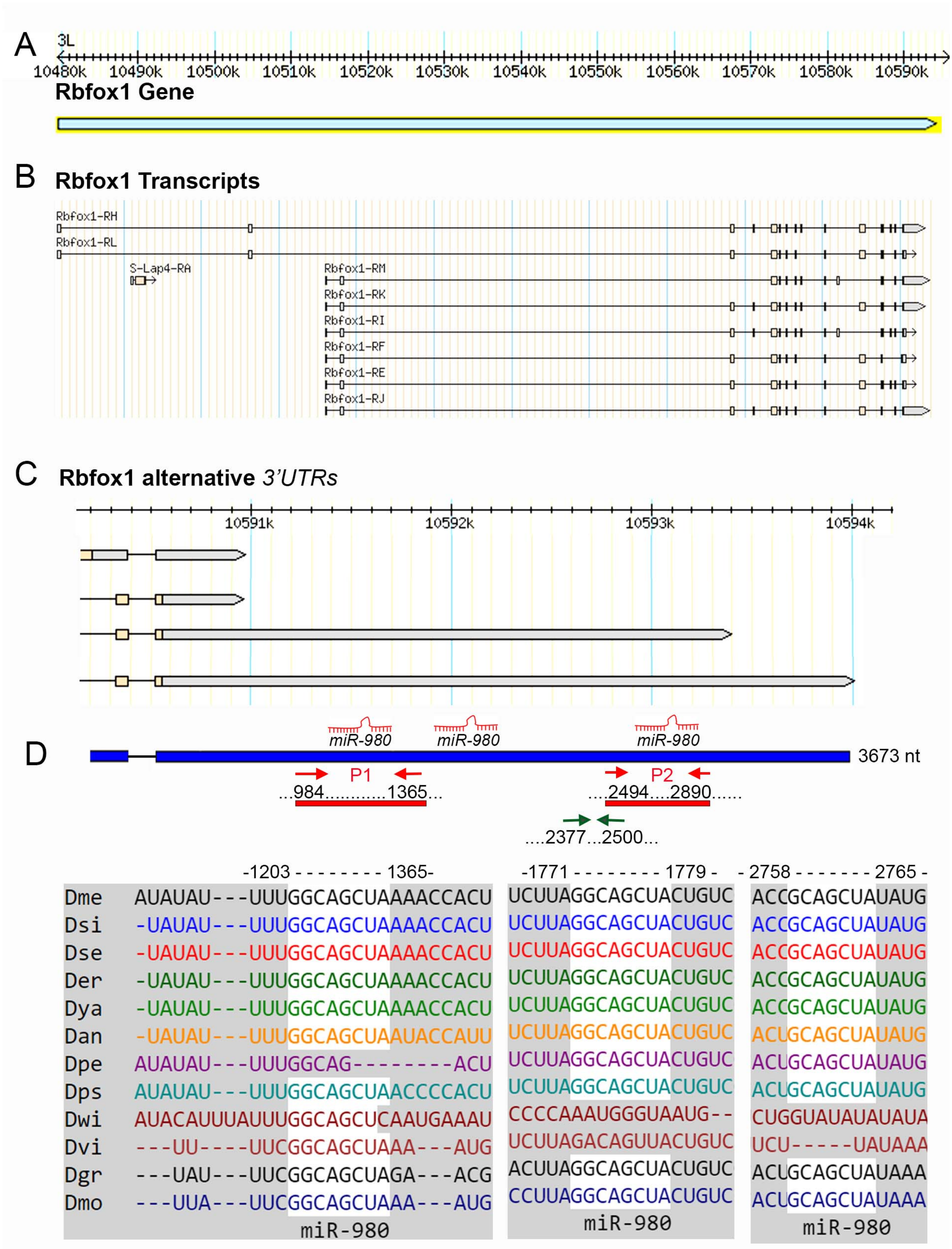

Supplementary Figure 2

Supplementary Figure 2. Model of *Rbfox1* gene, transcripts, and *3'UTRs*

A-B, *Rbfox1* gene is located on the 3rd chromosome and encodes multiple *Rbfox1* transcripts that are generated as a result of alternative splicing (http://flybase.org/reports/FBgn0052062.html). Based on recently published data, there is a possibility that additional *Rbfox1* isoforms exist [10].
C, *Rbfox1* transcripts differ by the choice of polyadenylation sites, which results in the appearance of *Rbfox1* mRNAs with alternative *3'UTRs*.
D, Note that only the extended *3'UTRs* have three evolutionarily conserved *miR-980* binding sites as predicted by TargetScanFly (http://www.targetscan.org/cgi-bin/targetscan/fly_12/view_gene.cgi?taxid=7227&gs=CG32062&showcnc=0&shownc=0#miR-980). Based on FlyBase annotations, different spliceoforms have distinct *3'UTRs*. For example, the RE isoform that was used for the overexpression studies has the shortest *3'UTR*; the RM isoform has the longest *3'UTR,* suggesting that it can be targeted by *miR-980*.
Primers used to generate P1 and P2 plasmids for the luciferase assay to test the ability of *miR-980* to target different parts of *Rbfox1 3'UTR* are shown by red arrows. Primers used in the RT-qPCR to test the levels of Rbfox1 mRNAs with the extended *3'UTR* are shown by dark green arrows. For primer sequences, refer to Materials and Methods.

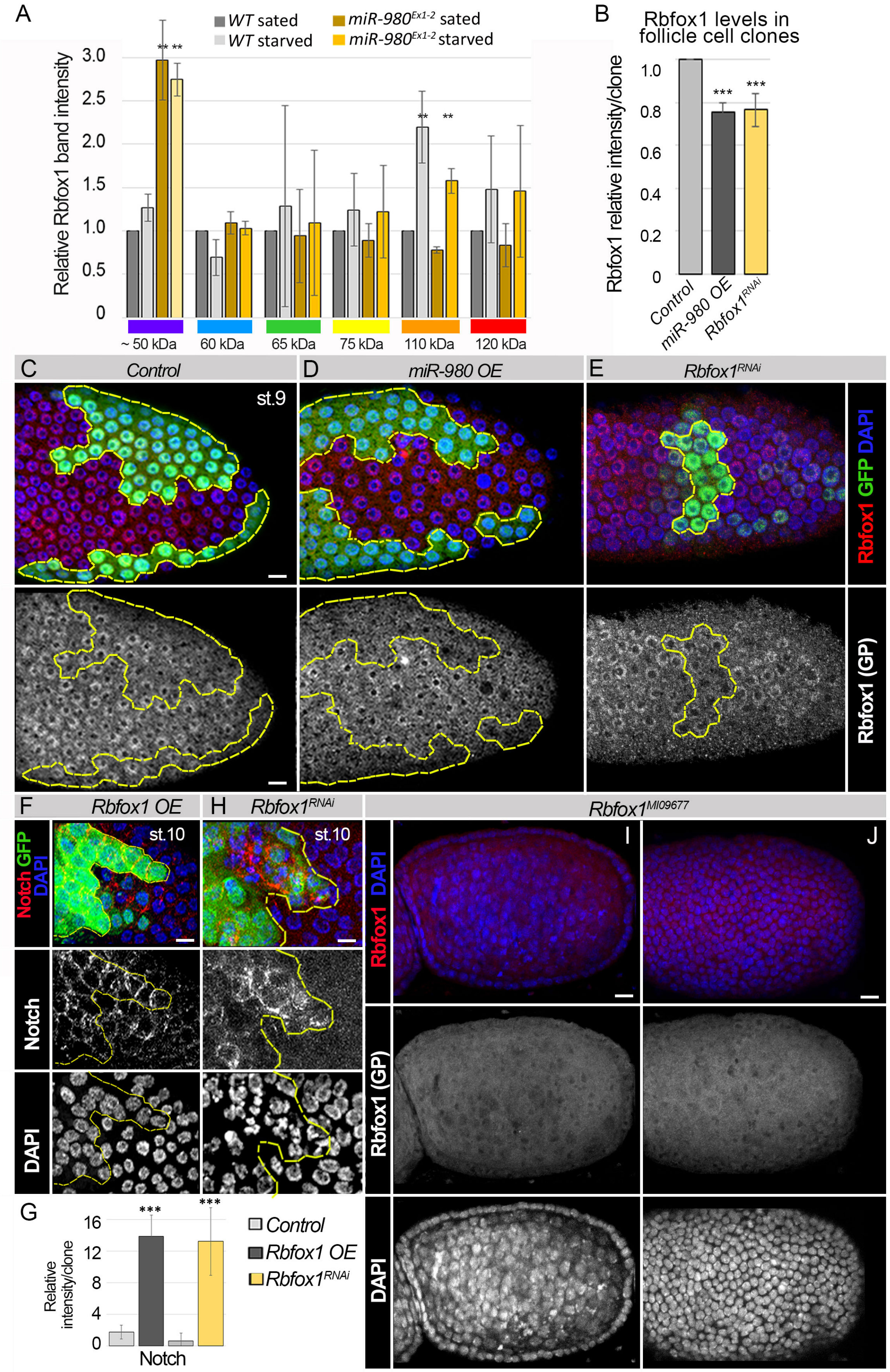

Supplementary Figure 3

Supplementary Figure 3. Rbfox1 levels depend on *miR-980* and stress

A, Bar graph shows relative protein levels for various Rbfox1 isoforms in sated and starved *wild type* and *miR-980* mutant ovaries presented as relative mean intensity of 3-5 biological replicates. For statistics, the two-tailed Student's t-test was applied, **$p<0.01$*. **B,** Relative Rbfox1 protein levels measured by the comparison of fluorescence intensity in follicle cell clones are decreased upon *miR-980* overexpression or *Rbfox1* downregulation via RNAi. **C-E**, Stage 9 egg chambers with *Control* (**C,** *hsFlp;; act>CD2>Gal4 UAS-GFP*), *miR-980*-overexpressing (**D,** *hsFlp; UAS-miR-980/+; act>CD2>Gal4 UAS-GFP*) and *Rbfox1 RNAi* (**E,** *hsFlp; UAS-Rbfox1^{RNAi}/+; act>CD2>Gal4 UAS-GFP*) clonal follicular epithelium cells marked with GFP. Yellow dashed lines outline clone contours. **D,** *miR-980* overexpression in the GFP-marked follicle cell clones downregulates Rbfox1 protein, implying cell-autonomous *miR-980*/Rbfox1 regulation during oogenesis. **F-G**, Levels of Notch signaling receptor are significantly increased in st.10 follicle cell clones with higher and lower Rbfox1 levels (**F** and **H,** respectively), when compared to the neighboring non-clonal cells. This suggests that the Notch receptor is not cleaved in a timely fashion, thus activation of Notch signaling, required for proper follicle cell differentiation, is defective in cells with abnormal Rbfox1 levels. **G,** Bar graph represents quantifications of antibody staining intensities for anti-Notch intracellular domain in *Control*, Rbfox1-expressing (*Rbfox1 OE*) and Rbfox1 knock-down (*Rbfox1^{RNAi}*) follicle cell clones, presented as relative to the intensity of a distal control follicle cell clone of the same size (n=11 clones for each genotype). **I-J,** Egg chamber of a hypomorphic *Rbfox1* mutant (*Rbfox1^{MI09677})* contains no nurse cells, instead it is filled with undifferentiated germline cells (possibly cystoblasts, DAPI, **I**) that are covered by follicular epithelium cells that fail to switch their mitotic cell cycle mode into the endocycle, as suggested by their increased numbers and small nuclei (DAPI, **J**), when compared to the endocycling follicle cells of same size egg chambers in **C-E**. Also, note that in *Rbfox1^{MI09677}* hypomorphic mutants, Rbfox1 staining is dramatically reduced, which confirms the specificity of anti-Rbfox1 (guinea pig) antibody. Quantifications of antibody staining intensities presented as AVE±SD relative to control. Student's t-test was applied for statistics in **A-B, H.** *$p\leq0.05$; **$p\leq0.01$; ***$p\leq0.001$. Images are maximum intensity projections of multiple z-slices. Scale bars 5μm.

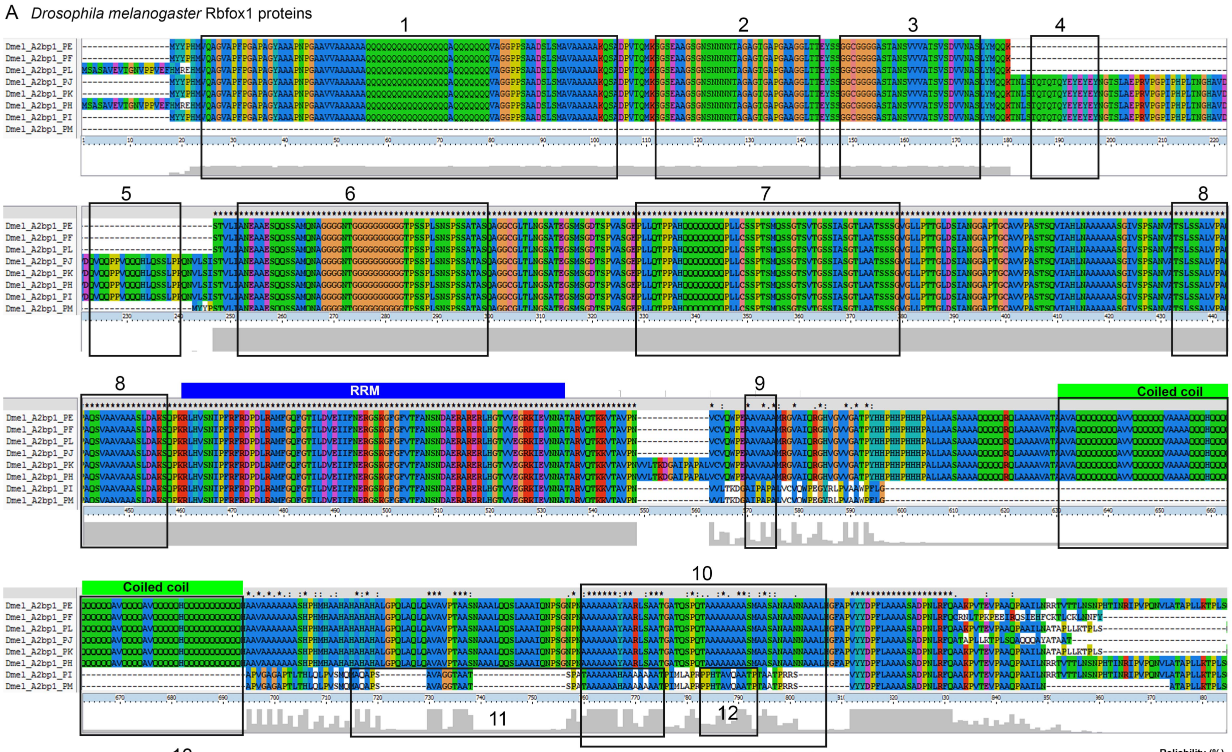

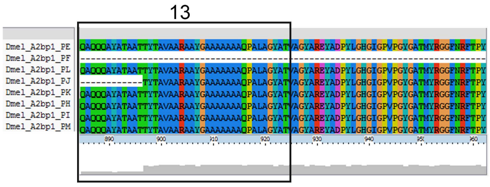

B

Isoform	PolyQ stretches in Rbfox1								
	Q_3	Q_4	Q_5	Q_6	Q_7	Q_8	Q_9	Q_{11}	Q_{16}
E	2	1	2	1	1	2	1	1	1
F	1	1	2	1	1	2	1	1	1
L	2	1	2	1	1	2	1	1	1
J	3	1	2	1	1	2	1	1	1
K	3	1	2	1	1	2	1	1	1
H	3	1	2	1	1	2	1	1	1
I	2	-	-	-	1	1	-	-	1
M	1	-	-	-	-	1	-	-	-

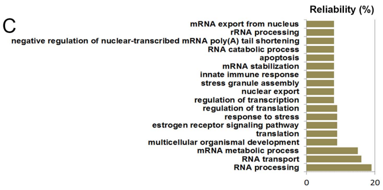

Supplementary Figure 4. *Drosophila* Rbfox1 protein contains multiple LCDs and polyQ stretches

A, Protein sequence comparisons of 8 annotated *D. melanogaster* Rbfox1 isoforms. *Drosophila* Rbfox1 protein contains one RRM (RNA recognition motif) and LCDs (low complexity sequence domains, black frames). The color of a symbol depends on the frequency of residue occurrence in the column and its type: BLUE >60% of hydrophobic (ACFHILMVWY), MAGENTA >50% with negative charge (DE), RED >60% with positive charge (KR), GREEN >50% Polar (STQN), PINK >85% Cysteines, ORANGE >85% Glycines, YELLOW >85% Prolines, CYAN >50% Aromatic (FYW) amino acids. **B**, Table shows number of polyQ stretches in different Rbfox1 isoforms. Q_3-Q_{16} indicates the number of glutamine repeats in each polyQ stretch. **C**, A bar graph shows GO protein functions for *Drosophila* Rbfox1.

Rbfox1-PE amino acid sequence

MYYPHMVQAGVAPFPGAPAGYAAAPNPGAAVVAAAAAAQQQQQQQQQQQQQQQQAQQQQQQQVAGGPPSAADSLSMAVAAAAAKQSADPVTQMKSGSEAAGSGN
SNNNNTAGAGTGAPGAAGGLTTEYSSGGCGGGGASTANSVVVATSVSDVVNASLYMQQKSTVLIANEAAESQQSSAMQNAGGGGNTGGGGGGGGGGTPSSPLSN
SPSSATASQAGGCGLTLNGSATEGSMSGDTSPVASGEPLLQTPPAHQQQQQQQQQPLLCSSPTSMQSSGTSVTGSSIASGTLAATSSSGVGLLPTTGLDSIANGG
APTGCAVVPASTSQVIAHLNAAAAAASGIVSPSANVATSLSSALVPAQSVAAVAAASLDAKSQPKRLHVSNIPFRFRDPDLRAMFGQFGTILDVEIIFNERGSK
GFGFVTFANSNDAERARERLHGTVVEGRKIEVNNATARVQTKKVTAVPNVCVQWPEAAVAAAMRGVAIQRGHVGVVGATPYHHPHHPHHHPALLAASAAAAQQQ
QQRQLAAAAVATAAVAQQQQQQQQAVVQQQQQQVAAAAQQQHQQQQQQQQQAVQQQQAVQQQQQHQQQQQQQQQQQHAAVAAAAAAAASHPHMHAAHAHAHAHAL
GPQLAQLQAVAVPTAASNAAALQQSLAAAIQNPSGNPNAAAAAAAYAARLSAATGATQSPQTAAAAAAAAASMAASANAANNAAALHGFAPVYYDPFLAAAASAD
PNLRFQAAKPVTEVPAAQPAAILNRRTVTTLNSNPHTINRIPVPQNVLATAPLLKTPLSQAQQQAYATAATTYTAVAARAAYGAAAAAAAQPALAGYATVAGYA
REYADPYLGHGIGPVPGYGATMYRGGFNRFTPY

Rbfox1-PE domains and their sequences

Mitochondrial Localization Signal YPHMV

Positively Charged AMPHIPHILICITY Region YPHMVQAGVAPF

LCD1 VQAGVAPFPGAPAGYAAAPNPGAAVVAAAAAAQQQQQQQQQQQQQQQQAQQQQQQQVAGGPPSAADSLSMAVAAAAAKQSA

LCD2 SGSEAAGSGNSNNNNTAGAGTGAPGAAGGLTT

LCD3 GGCGGGGASTANSVVVATSVSDVVNAS

LCD6 ANEAAESQQSSAMQNAGGGGNTGGGGGGGGGGTPSSPLSNSPSSATAS

LCD7 PLLQTPPAHQQQQQQQQQPLLCSSPTSMQSSGTSVTGSSIASGTLAATSSSG

LCD8 SLSSALVPAQSVAAVAAASLDAKS

RRM RLHVSNIPFRFRDPDLRAMFGQFGTILDVEIIFNERGSKGFGFVTFANSNDAERARERLHGTVVEGRKIEVN

LCD9 AAVAAA

Coiled Coil AAVAQQQQQQQQAVVQQQQQQVAAAAQQQHQQQQQQQQQAVQQQQAVQQQQQHQQQQQQQQQQQH

LCD10 AAAAAAAYAARLSAATGATQSPQTAAAAAAAAASMAASANAANNAAA

LCD13 QAQQQAYATAATTYTAVAARAAYGAAAAAAAQPALAGYAT

Rbfox1-PE protein domain scheme

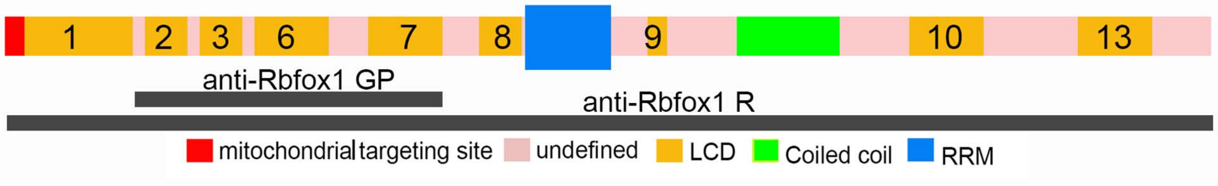

Supplementary Figure 5. Sequence and predicted protein domains of Rbfox1-PE isoform that was used in the overexpression studies

Yellow blocks indicate LCDs, among which coiled coil region is marked by green, cyan marks RRM predicted by SMART. The sequence marked in red corresponds to the mitochondrial localization signal. The sequence underlined by the bold line corresponds to residues 84-186AA in the Rbfox1-PE isoform used to generate polyclonal guinea pig anti-Rbfox1 antibodies (Rbfox1 GP) [11]. To generate polyclonal rabbit anti-Rbfox1 antibodies (Rbfox1 R), the full length Rbfox1-PE isoform was used [8]. For protein sequence alignment, ClustalX 2.1 was used, and for protein sequence analysis and domain identification, Simple Modular Architecture Research Tool (SMART) [12] and MitoFates [13] were applied.

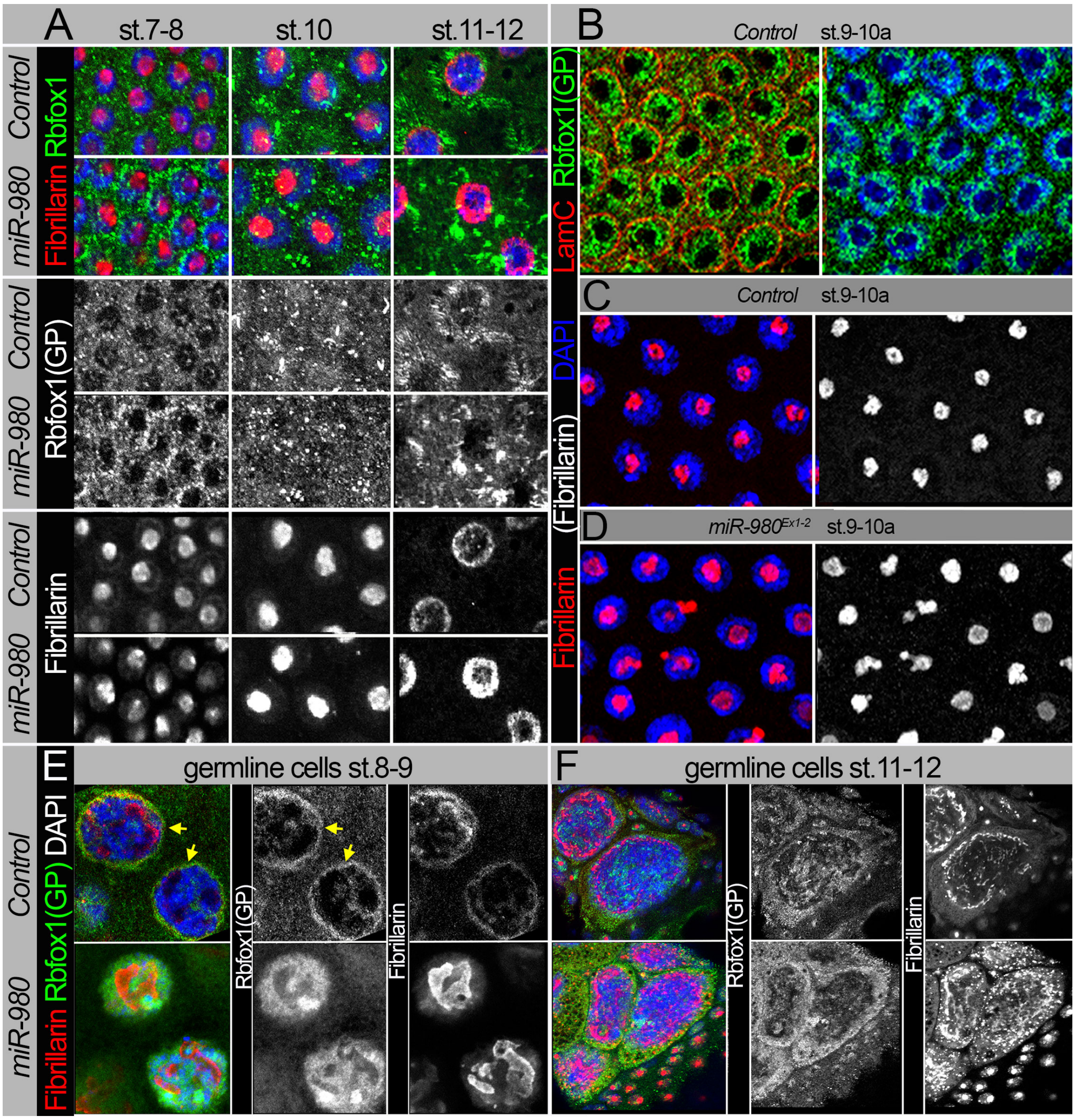

Supplementary Figure 6. Rbfox1 expression levels are increased in the germline and soma of *miR-980* mutants

A, Rbfox1 protein has a dynamic expression pattern in differentiating follicular epithelial cells in st.7-12 egg chambers. Depending on the stage (st 7-10 – endocycle, st.11-12 – amplification), it shows predominantly cytoplasmic and sometimes nuclear granular patterns and can be detected in small foci, enlarged granules, or even short fibers (lower panels). The Rbfox1 expression pattern is affected by *miR-980* loss. **B**, In follicle cell nuclei prior to the amplification stage, Rbfox1 is associated with chromatin and enriched in the granular component of the nucleolus, which is a ring-like structure around the nucleolar fibrillar center, marked by Fibrillarin. **C-D,** Nucleolar appearance is altered in *miR-980* mutants (**D**) in comparison to controls (**C**). **E-F**, In the endoreplicating germline nurse cells nuclei, Rbfox1 expression pattern is developmental stage-dependent. In the absence of *miR-980*, Rbfox1 pattern and the nucleolus appearance are changed. Note Rbfox1 localization to the nuage (yellow arrows).

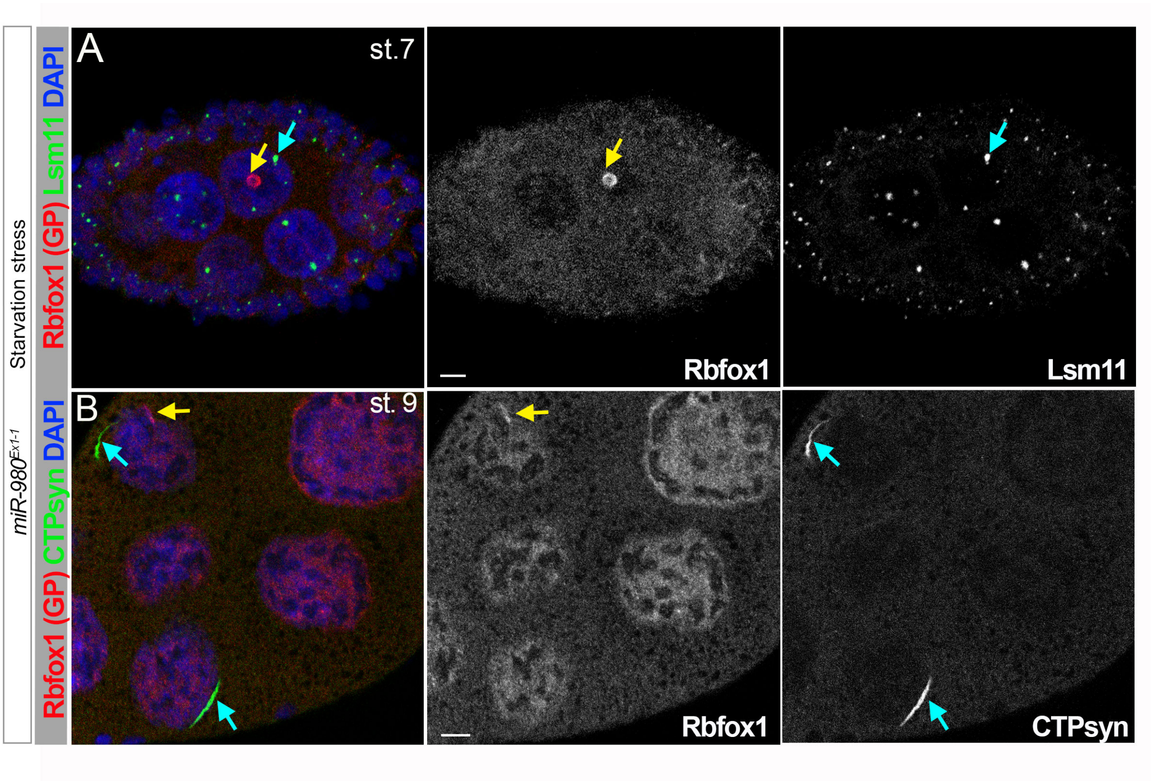

Supplementary Figure 7. Rbfox1 does not colocalize with histone locus body and cytoophidia

A, Histone locus body that contains factors necessary for processing of histone pre-mRNAs is marked by Lsm11 [14]. Note that Rbfox1 and Lsm11 do not co-stain. **B,** Rbfox1 does not co-localize with cytoophidia, a filamentous structure formed by CTP synthase [15,16].

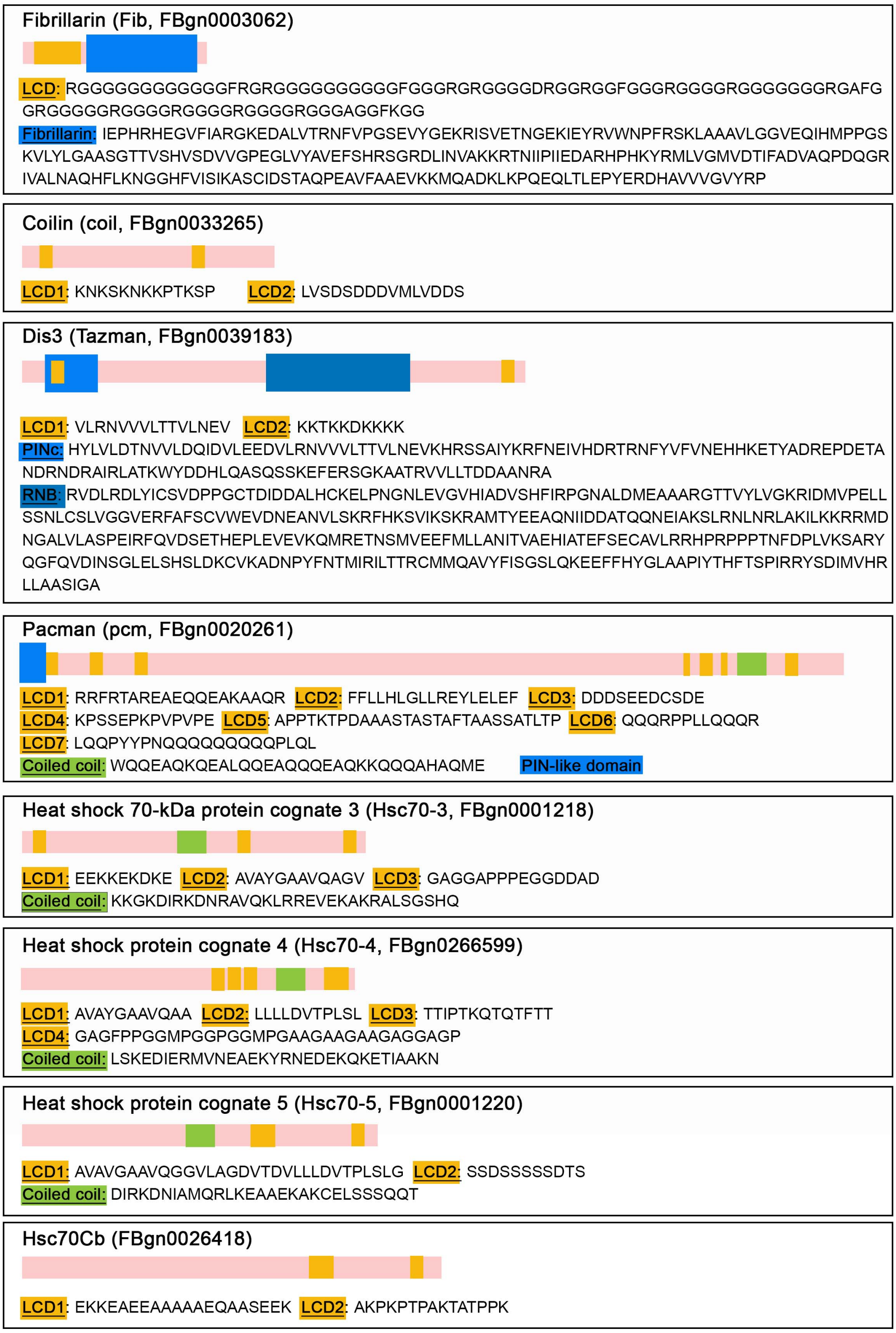

Supplementary Figure 8. Proteins detected to associate with Rbfox1 contain multiple LCDs

Protein domain structure predicted by SMART database and the amino acid sequences of the domains are shown. Yellow blocks indicate LCDs, among which a coiled coil region is marked by green. Blue blocks indicate other detected functional domains.

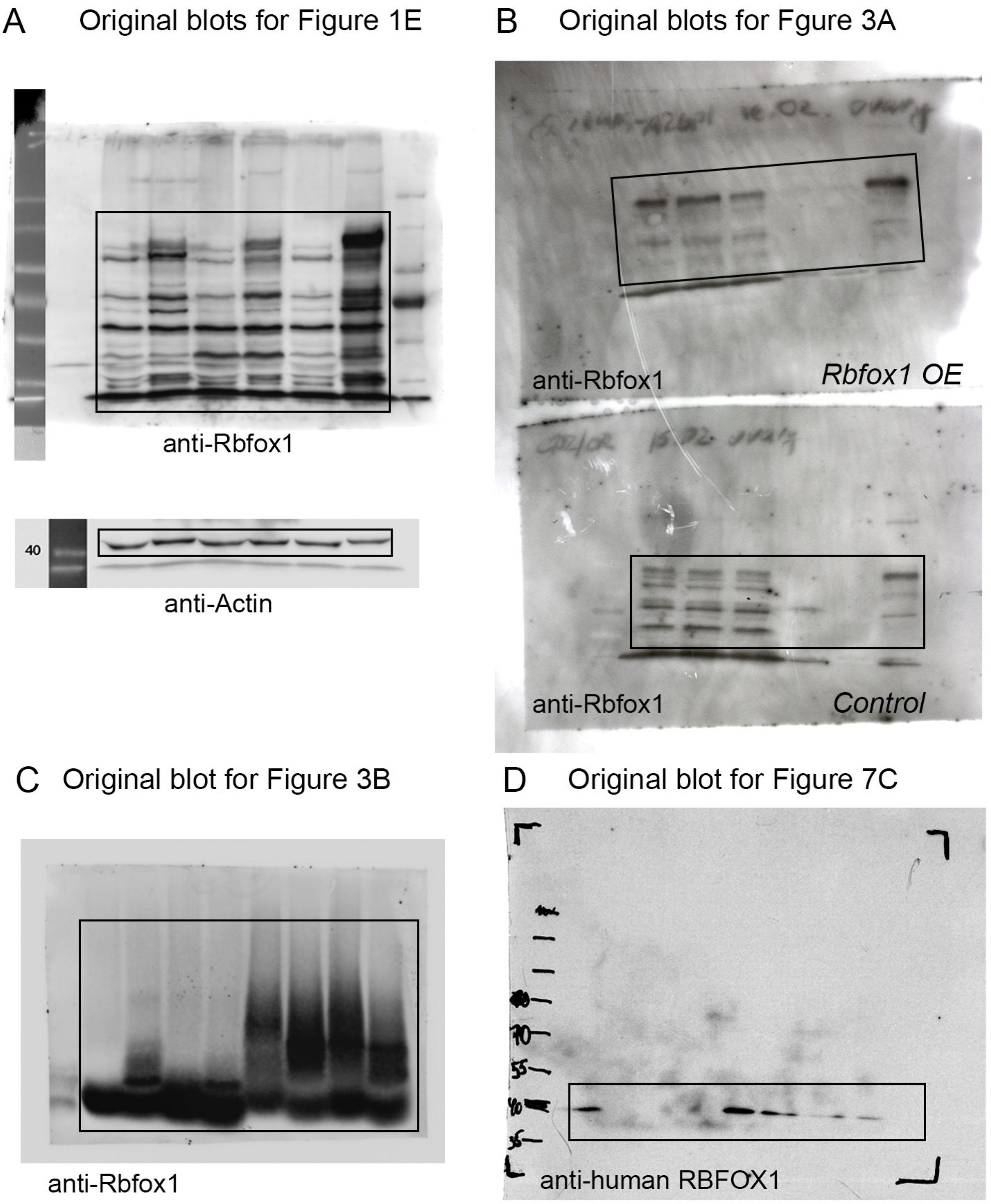

Supplementary Figure 9. Uncropped western blots
Black boxes highlight the area displayed in the corresponding Figure.

Supplementary References

1 Ruby, J. G. *et al.* Evolution, biogenesis, expression, and target predictions of a substantially expanded set of Drosophila microRNAs. *Genome research* **17**, 1850-1864, doi:10.1101/gr.6597907 (2007).

2 Lewis, B. P., Burge, C. B. & Bartel, D. P. Conserved seed pairing, often flanked by adenosines, indicates that thousands of human genes are microRNA targets. *Cell* **120**, 15-20, doi:10.1016/j.cell.2004.12.035 (2005).

3 Marrone, A. K., Edeleva, E. V., Kucherenko, M. M., Hsiao, N. H. & Shcherbata, H. R. Dg-Dys-Syn1 signaling in Drosophila regulates the microRNA profile. *BMC Cell Biol* **13**, 26, doi:10.1186/1471-2121-13-26 (2012).

4 Chen, Y. W. *et al.* Systematic study of Drosophila microRNA functions using a collection of targeted knockout mutations. *Developmental cell* **31**, 784-800, doi:10.1016/j.devcel.2014.11.029 (2014).

5 Schertel, C., Rutishauser, T., Forstemann, K. & Basler, K. Functional characterization of Drosophila microRNAs by a novel in vivo library. *Genetics* **192**, 1543-1552, doi:10.1534/genetics.112.145383 (2012).

6 Bajpai, R., Sambrani, N., Stadelmayer, B. & Shashidhara, L. S. Identification of a novel target of D/V signaling in Drosophila wing disc: Wg-independent function of the organizer. *Gene expression patterns : GEP* **5**, 113-121, doi:10.1016/j.modgep.2004.05.005 (2004).

7 Buszczak, M. *et al.* The carnegie protein trap library: a versatile tool for Drosophila developmental studies. *Genetics* **175**, 1505-1531, doi:10.1534/genetics.106.065961 (2007).

8 Usha, N. & Shashidhara, L. S. Interaction between Ataxin-2 Binding Protein 1 and Cubitus-interruptus during wing development in Drosophila. *Developmental biology* **341**, 389-399, doi:10.1016/j.ydbio.2010.02.039 (2010).

9 Greene, J. C. *et al.* Mitochondrial pathology and apoptotic muscle degeneration in Drosophila parkin mutants. *Proceedings of the National Academy of Sciences of the United States of America* **100**, 4078-4083, doi:10.1073/pnas.0737556100 (2003).

10 Guven-Ozkan, T. *et al.* MiR-980 Is a Memory Suppressor MicroRNA that Regulates the Autism-Susceptibility Gene A2bp1. *Cell reports* **14**, 1698-1709, doi:10.1016/j.celrep.2016.01.040 (2016).

11 Tastan, O. Y., Maines, J. Z., Li, Y., McKearin, D. M. & Buszczak, M. Drosophila ataxin 2-binding protein 1 marks an intermediate step in the molecular differentiation of female germline cysts. *Development* **137**, 3167-3176, doi:10.1242/dev.050575 (2010).

12 Letunic, I., Doerks, T. & Bork, P. SMART: recent updates, new developments and status in 2015. *Nucleic Acids Res* **43**, D257-260, doi:10.1093/nar/gku949 (2015).

13 Fukasawa, Y. *et al.* MitoFates: improved prediction of mitochondrial targeting sequences and their cleavage sites. *Mol Cell Proteomics* **14**, 1113-1126, doi:10.1074/mcp.M114.043083 (2015).

14 Nizami, Z. F., Deryusheva, S. & Gall, J. G. Cajal bodies and histone locus bodies in Drosophila and Xenopus. *Cold Spring Harb Symp Quant Biol* **75**, 313-320, doi:10.1101/sqb.2010.75.005 (2010).

15 Liu, J. L. Intracellular compartmentation of CTP synthase in Drosophila. *J Genet Genomics* **37**, 281-296, doi:10.1016/S1673-8527(09)60046-1 (2010).

16 Noree, C., Sato, B. K., Broyer, R. M. & Wilhelm, J. E. Identification of novel filament-forming proteins in Saccharomyces cerevisiae and Drosophila melanogaster. *J Cell Biol* **190**, 541-551, doi:10.1083/jcb.201003001 (2010).
